# Comparative Hepatotoxicity of Fluconazole, Ketoconazole, Itraconazole, Terbinafine, and Griseofulvin in Rats

**DOI:** 10.1155/2017/6746989

**Published:** 2017-02-05

**Authors:** Star Khoza, Ishmael Moyo, Denver Ncube

**Affiliations:** ^1^Department of Clinical Pharmacology, College of Health Sciences, University of Zimbabwe, Harare, Zimbabwe; ^2^Department of Anatomy, College of Health Sciences, University of Zimbabwe, Harare, Zimbabwe

## Abstract

Oral ketoconazole was recently the subject of regulatory safety warnings because of its association with increased risk of inducing hepatic injury. However, the relative hepatotoxicity of antifungal agents has not been clearly established. The aim of this study was to compare the hepatotoxicity induced by five commonly prescribed oral antifungal agents. Rats were treated with therapeutic oral doses of griseofulvin, fluconazole, itraconazole, ketoconazole, and terbinafine. After 14 days, only ketoconazole had significantly higher ALT levels (*p* = 0.0017) and AST levels (*p* = 0.0008) than the control group. After 28 days, ALT levels were highest in the rats treated with ketoconazole followed by itraconazole, fluconazole, griseofulvin, and terbinafine, respectively. The AST levels were highest in the rats treated with ketoconazole followed by itraconazole, fluconazole, terbinafine, and griseofulvin, respectively. All drugs significantly elevated ALP levels after 14 days and 28 days of treatment (*p* < 0.0001). The liver enzyme levels suggested that ketoconazole had the highest risk in causing liver injury followed by itraconazole, fluconazole, terbinafine, and griseofulvin. However, histopathological changes revealed that fluconazole was the most hepatotoxic, followed by ketoconazole, itraconazole, terbinafine, and griseofulvin, respectively. Given the poor correlation between liver enzymes and the extent of liver injury, it is important to confirm liver injury through histological examination.

## 1. Introduction

In 2013, the United States Food and Drug Administration (US FDA) and the European Medicines Agency's Committee on Medical Products for Human Use (EMA-CHMP) concurrently issued safety warnings and limited the use of oral ketoconazole because of its association with increased risk of inducing hepatic injury, risk of drug interactions, and increased risk of adrenal insufficiency [1, 2]. The two agencies recommended that ketoconazole should be used “only when alternative antifungal therapies are not available or tolerated.” In addition to the safety warning, the FDA issued another directive recommending that drug companies and researchers should avoid using oral ketoconazole in drug interaction studies [3]. The regulatory safety warnings on oral ketoconazole have serious implications on its use in the clinical and drug development settings. Ketoconazole has been widely used for more than three decades in the treatment of fungal infections and has been the principal prototype human cytochrome P450 3A inhibitor in drug interaction studies and drug metabolism research during drug development [4–6].

The link between ketoconazole and hepatotoxicity is well established [7–10]. However, for a long time the evidence suggested that the hepatotoxicity was mild, rarely fatal, and reversible upon discontinuation of the drug [7, 8, 10]. An estimated prevalence of serious hepatotoxicity of one in 15,000 patients was reported in the United Kingdom in the first decade of oral ketoconazole market authorization [10]. Incidence data on ketoconazole induced hepatotoxicity is scarce. In a randomized controlled study, subclinical hepatic dysfunction was observed in 17.5% of patients treated with ketoconazole while none of the patients treated with griseofulvin had evidence of hepatic dysfunction [11]. By contrast, a recent meta-analysis of 204 studies reported an overall incidence of ketoconazole-associated hepatotoxicity of between 3.6% and 4.2% [12].

Although the hepatotoxicity of antifungal agents is well established [9, 13–19], their relative hepatotoxicity has not been extensively evaluated [20]. Two epidemiological studies reported contrasting findings regarding the relative hepatotoxicity of antifungal agents [21, 22]. One of the studies cited by the FDA in its regulatory decision reported that ketoconazole was associated with the highest relative risk (RR = 228; 95% CI: 33.9–933.0) when compared to nonusers, followed by itraconazole (RR = 17.7; 95% CI: 2.6–72.6) and terbinafine (RR = 4.2; 95% CI: 0.2–24.9) [21]. This study included a cohort of 69,830 patients in the United Kingdom who had received at least one prescription for fluconazole, griseofulvin, itraconazole, ketoconazole, or terbinafine between 1991 and 1996. Of the 69,830 patients included in the study, only 1052 received ketoconazole. The incidence rates of hepatotoxicity were highest in patients treated with ketoconazole (19.0 per 10,000), followed by itraconazole (1.0 per 10,000) and terbinafine (0.7 per 10,000) [21]. In contrast to the study by García Rodrǐguez et al. (1999), Kao and associates reported the highest incidence rate of drug-induced liver injury of 31.6 per 10,000 patients in individuals who received fluconazole, compared to 4.9 for ketoconazole, 4.3 for griseofulvin, 3.6 for itraconazole, and 1.6 for terbinafine [22]. The study included 90,847 patients in Taiwan who received oral antifungal agents between 2002 and 2008. Of these patients, 57,321 received oral ketoconazole [22].

Based on the currently available evidence, it is uncertain which antifungal agent poses the greatest risk of hepatotoxicity. The small number of cases in the two epidemiological studies that reported on the relative hepatotoxicity of antifungal agents limits the interpretation of their findings [21, 22]. The findings by García Rodrǐguez et al. (1999) were based on 16 cases of acute liver injury [21]. Of these 16 cases, five occurred during current use of oral antifungal agents: two were using ketoconazole, two were using itraconazole, and one was using terbinafine. Out of the ten remaining cases, only one had a history of using an antifungal agent while the other nine cases occurred before the use of any antifungal agent. Similarly, the study by Kao et al. (2014) was based on only 52 cases of drug-induced liver injury [22]. Of the 52 cases, 28 used ketoconazole, 14 were of fluconazole, 8 were of griseofulvin, 3 were of itraconazole, and 2 were of terbinafine. In addition to the failure by these two epidemiologic studies to provide conclusive evidence regarding the antifungal agent with the greatest risk of hepatotoxicity, few head-to-head experimental studies have evaluated the relative hepatotoxicity of oral antifungal agents in clinical settings or using animal models [11, 23–25]. Furthermore, the higher number of cases of liver injury reported with ketoconazole than fluconazole might be related to the higher number of prescriptions for ketoconazole than fluconazole. Given the implications of the safety warnings issued in 2013 on the use of ketoconazole in clinical settings and during drug development research, there is need for experimental studies that evaluate the relative hepatotoxicity of azole antifungal agents. The objective of this study was to compare the hepatotoxicity effects of the five commonly prescribed oral antifungal agents (ketoconazole, fluconazole, itraconazole, terbinafine, and griseofulvin). We hypothesized that fluconazole is more hepatotoxic than ketoconazole based on histological examination.

## 2. Materials and Methods

### 2.1. Materials

All biochemical kits for alanine aminotransferase (ALT), aspartate amino transferases (AST), and alkaline phosphatase (ALP) were sourced from Beckman Coulter Inc. (California, USA). Terbinafine (Lamisil®; batch number U0638; marketed by Novartis Pharma Ltd., United Kingdom), itraconazole (Canditral®; batch number 01141282; marketed by Glenmark Pharmaceuticals Ltd., India), griseofulvin (Griseon®; batch number 178046; marketed by Plus Five Pharmaceuticals Ltd., Zimbabwe), fluconazole (Flumyc-200®; batch number AHP054014; marketed by Ipca Laboratories Ltd., India), and ketoconazole (Nizol®; batch number 13312; marketed by Intas Pharmaceuticals Ltd., India) were all sourced from a local pharmaceutical wholesaler. Standard diet pellets were obtained from National Foods Pvt. Ltd., Zimbabwe. Formaldehyde (37% solution), paraffin wax, haematoxylin, eosin, and other standard laboratory chemicals were sourced from Sigma-Aldrich (United Kingdom). Blood collection tubes (Vacuette® Z serum clot activator tubes) were sourced from Greiner Bio-One (United Kingdom). Microcentrifuge tubes (LW2075; batch number 110488) were sourced from Alpha Laboratories (Hampshire, United Kingdom).

### 2.2. Animals and Dosing Procedures

Sixty-six 6-week-old male Sprague Dawley rats weighing 180–200 g were adapted to laboratory conditions for five days before experimentation. The rats were housed in plastic cages in groups of six with wood shavings as bedding under a 12-hour light/12-hour dark cycle. The rats were maintained in a conventional animal house with an ambient temperature of 25 ± 2°C and were given commercial standard diet rat pellets and tap water ad libitum. Ethical clearance to conduct the study was obtained from the Joint Parirenyatwa Hospital and College of Health Sciences Research Ethics Committee (approval number: JREC/328/14). The animals were handled and treated following the principles outlined in the “Guide for the Care and Use of Laboratory Animals” prepared by the National Academy of Sciences and published by the National Institutes of Health (NIH publication 86-23 Rev. 1985).

The rats were divided into eleven groups (each group with six rats) including the control group. The rats in the control group were sacrificed one day before drug administration (day 0) in the active treatment groups. Five groups of rats received a daily single oral antifungal agent dose for 14 days. The other five groups of rats received a daily single oral antifungal agent dose for 28 days. The intragastric method (oral gavage) was used during drug administration. The treatment interventions were 20 mg/kg fluconazole, 50 mg/kg griseofulvin, 20 mg/kg ketoconazole, 20 mg/kg itraconazole, and 25 mg/kg terbinafine. Antifungal agents are frequently prescribed for two weeks or four weeks for most systemic and topical fungal infections. The equivalent doses in rats for common adult dose ranges for systemic and topical infections for the antifungal agents are as follows: fluconazole (10–20 mg/kg), griseofulvin (25–50 mg/kg), ketoconazole (10–20 mg/kg), itraconazole (10–40 mg/kg), and terbinafine (10–25 mg/kg). A suspension of each drug in distilled water was prepared two hours prior to administration. The doses were calculated using the formula provided by the US FDA (i.e., animal dose = clinical human dose × conversion factor for rats [6.2]) [26].

### 2.3. Sampling and Biochemical Assays

Blood samples were collected on three different occasions on days 0, 15, and 29, using the cardiac puncture method. Day 0 was the day before initial drug administration. Blood samples collected from rats sacrificed on day 0 were for the determination of baseline liver enzyme levels in the clan of the rats. Day 15 was defined as the day after the rats had completed 14-day courses of drug treatment, that is, 24 hours after last dose. Day 29 was defined as the day after the rats had completed 28-day courses of treatment. Blood samples (4.0 ml) were collected using red top (black ring) vacutainers (6.0 ml Vacuette® Z serum clot activator tubes; Greiner Bio-One Ltd., UK) and were left to clot for 30 minutes before being spun down. Blood samples were spun down at 1200 rpm for 10 minutes and the serum transferred to 1.5 ml plastic microcentrifuge tubes (Alpha Laboratories, UK). The serum was then stored at −18°C up to the day of analysis (at most 7 days). The Beckman AU680® chemistry analyser was used to determine plasma levels of ALP, AST, and ALT. Aspartate transaminase (AST) and alanine transaminase (ALT) activity in serum were assayed using a procedure based on the method developed by Wróblewski and Ladue [27–29]. Alkaline phosphatase (ALP) activity in serum was assayed by a procedure based on the method developed by Bowers Jr. and McComb [30].

### 2.4. Histological Examination

All the animals were sacrificed humanly under chloroform anaesthesia at the end of each treatment period. Livers were removed and fixed in 10% formalin for 24 hours. The whole liver tissue samples were then put in an automated tissue processer Leica TP10202 for 24 hours for dehydration using alcohol and clearing using xylol. The liver samples were then embedded in paraffin wax and cut into sections of 5 *µ*m thickness, mounted on clean glass slides coated with Mayer's egg albumin, and were stained with hematoxylin and eosin (H&E). Liver sections containing the central venule were used to make comparisons across treatment groups. Light microscopy (Motic BA210®) was used to generate photomicrographs during histological examinations. All histologic examinations were performed by the same histologist (DN).

### 2.5. Statistical Analysis

Data are presented as the mean value ± standard error of the mean (SEM). Comparisons among multiple groups were done using one way ANOVA followed Tukey's post hoc test as appropriate. Two group comparisons were done using Student's *t*-test. Kruskal-Wallis test and Mann–Whitney test were used as appropriate whenever the normality assumption was violated. The significance level was set at *α* = 0.05. Statistical analysis was carried out using Graph Pad® Prism Version 6.0 for Windows (California, USA).

## 3. Results

### 3.1. Biochemical Findings

After 14 days of treatment, only ketoconazole had significantly higher ALT levels (*p* = 0.0017) and AST levels (*p* = 0.0008) than the control group. The ALT and AST levels were highest in rats treated with ketoconazole, followed by fluconazole, itraconazole, terbinafine, and griseofulvin. ALT levels in rats treated with ketoconazole were significantly higher than the levels in rats treated with griseofulvin (*p* = 0.0066) and terbinafine (*p* = 0.0074). Similarly, AST levels in rats treated with ketoconazole were significantly higher than the levels in rats treated with griseofulvin (*p* = 0.0076) and terbinafine (*p* = 0.0109). However, no significant differences in ALT and AST levels were observed between ketoconazole and fluconazole and between ketoconazole and itraconazole (*p* > 0.05). All drugs had significantly higher ALP levels than the control group after 14 days of treatment (*p* < 0.0001). Rats treated with terbinafine had the highest ALP levels, followed by those treated with itraconazole, fluconazole, griseofulvin, and ketoconazole, respectively.  Table 1 shows the effect of the antifungal agents on liver enzymes in rats after 14 days of treatment.

After 28 days of treatment, all the drugs had significantly higher AST levels compared to the control group (*p* < 0.001). The AST levels were highest in the rats treated with ketoconazole followed by itraconazole, fluconazole, terbinafine, and griseofulvin, respectively. All drugs, except terbinafine, had significantly higher ALT levels compared to the control group after 28 days of treatment (*p* < 0.05). The ALT levels were highest in the rats treated with ketoconazole followed by itraconazole, fluconazole, griseofulvin, and terbinafine, respectively. All drugs had significantly higher ALP levels than the control group after 28 days of treatment (*p* < 0.001). Fluconazole caused the highest ALP levels, followed by itraconazole, terbinafine, ketoconazole, and griseofulvin, respectively.  Table 2 shows the effect of the antifungal agents on liver enzymes in rats after 28 days of treatment.

**Figure 1:**
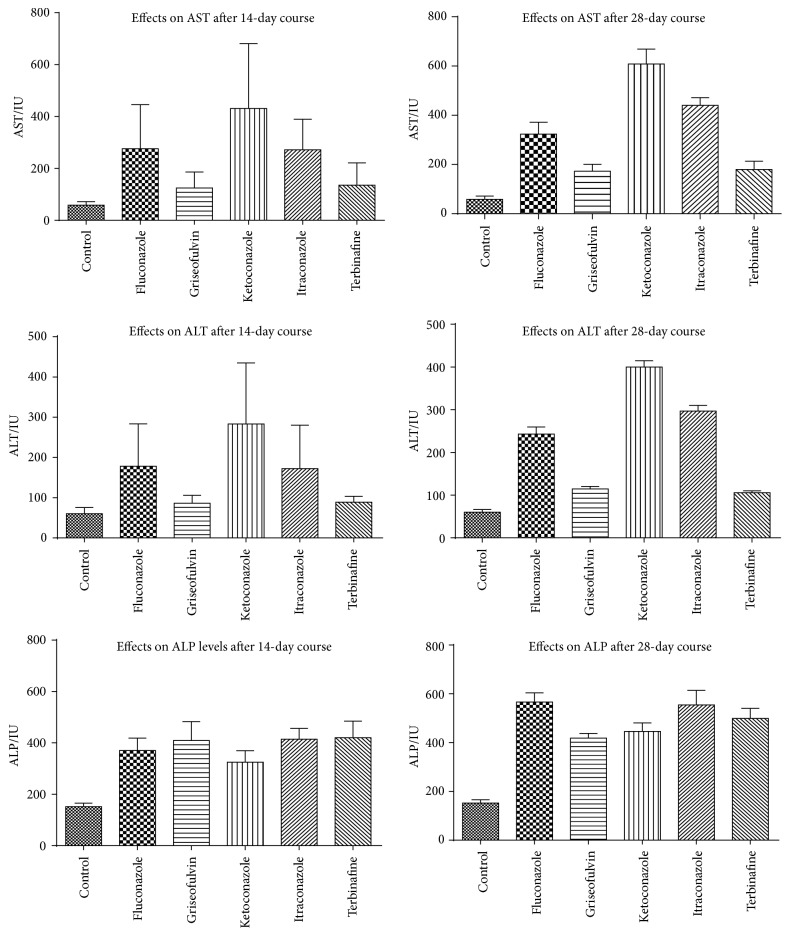
Serum levels of AST, ALT, and ALP, for the control group and the groups that received 14- and 28-day courses of fluconazole, griseofulvin, ketoconazole, itraconazole, and terbinafine.

AST levels after 28 days of treatment with itraconazole were significantly higher than the levels after 14 days of treatment (*p* = 0.0071). Similarly, treatment with itraconazole (*p* = 0.0226), terbinafine (*p* = 0.0434), and griseofulvin (*p* = 0.0190) for 28 days resulted in higher ALT levels than treatment for 14-day courses, respectively. Furthermore, there were significant duration-dependent elevations in ALP levels after treatment with fluconazole (*p* = 0.0009), ketoconazole (*p* = 0.0004), itraconazole (*p* = 0.0009), and terbinafine (*p* = 0.0300) for 28 days compared with treatments for 14 days.  Figure 1 shows a comparison of liver enzymes after 14 days and 28 days of treatment.

### 3.2. Histopathological Findings

**Figure 2:**
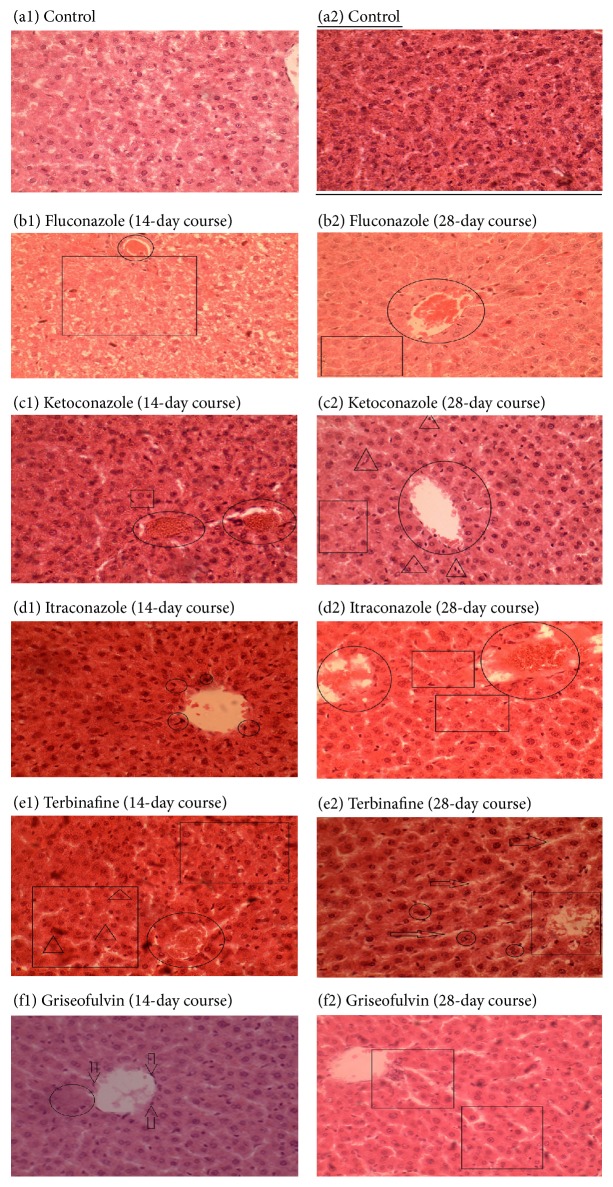
Photomicrographs of liver sections of the control group (a) and after 14- and 28-day courses of fluconazole (b), ketoconazole (c), itraconazole (d), terbinafine (e), and griseofulvin (f). (a) Normal lobulation with clear outlines, normal cells with visible outlines, and single nuclei. (b) In 14-day plate the rectangular area shows a marked reduction of cell numbers in the perivenular area. Circular areas indicate infiltration of venules by leukocytes. 28-day course plate shows reduced cellular density similar to 14-day plate. (c) Rectangular area indicates reduced cell density; circular areas indicate perivenular region with minor necrotic figures, that is, dark spots on plate. Triangular region indicates scattered necrotic foci (28-day course). (d) Minor venular distortion indicated by the circular demarcations here; triangular areas show apoptotic cells. Rectangular area shows reduced cell density. (e) Rectangular area shows paucity of cells; circular areas show clustering of cells which is a possible indication of stress. (f) Rectangular areas show a reduction in cell numbers and tissue striations (28 d). Perivenular cell paucity is indicated by the circular area on the plate (14 d). Also apparent are necrotic foci around the central venue (arrows). All plates are ×200 magnification.

There were no gross pathological changes observed by naked eye examination.  Figure 2 shows photomicrographs of the livers of the control and after treatment with the antifungal agents. Light microscopic examination of livers of control rats showed normal lobulation with clear outlines, normal Kupffer cells with distinct cell boundaries and clearly visible nuclei, no infiltration of central venules by leukocytes, and lack of mitotic figures (Figure 2(a)). Treatment with fluconazole resulted in the most severe damage compared to all the groups. A reduction in cell nuclear density in centrilobular, severe hepatocyte degeneration, severe inflammation and necrosis, granuloma, and bile duct hyperplasia were observed after fluconazole treatment (Figure 2(b)). Although there was no significant difference in cell death between the 14- and 28-day courses, in the 28-day course the tissue exhibited minor indications of recovery with the normal lobulation appearing. Infiltration of central venules by leukocytes and nuclei of Kupffer cell were less after 28 days of treatment.

Ketoconazole caused the same level of hepatocyte degeneration, inflammation, and necrosis as that observed with fluconazole (Figure 2(c)). However, there were fewer granulomas and less severe bile duct hyperplasia during treatment with ketoconazole than with fluconazole. Venular infiltration and hepatic parenchymal invasion, in addition to centrilobular degeneration, was observed during treatment with ketoconazole. By contrast, mitotic figures, cell atrophy, and fewer nuclei were more profound compared to other groups. The severity of hepatic damage increased slightly during the 28-day course with more necrotic cells being observed than during the 14-day course. However, leukocyte infiltration was moderately less after 28 days of treatment than over the 14-day course.

In the group treated with itraconazole, most notable features were leukocytes infiltration of the central venules and mitotic figures, which worsened after 28 days of treatment compared to 14 days of treatment (Figure 2(d)). Terbinafine caused mild hepatic damage during both courses, with few mitotic figures and minor central venule infiltration by leukocytes observed (Figure 2(e)). No significant duration-dependent cell damage was observed during treatment with terbinafine. Griseofulvin caused mild hepatic damage, inflammation, centrilobular necrosis, and central venule infiltration by leukocytes (Figure 2(f)). No significant duration-dependent cell damage was observed during treatment with griseofulvin and this group caused the least hepatic damage. Based on the histological observations, fluconazole caused the worst hepatic damage followed by ketoconazole, itraconazole, terbinafine, and griseofulvin, respectively. The summary of histopathological findings is presented in Table 3.

## 4. Discussion

The purpose of the present study was to compare the hepatotoxicity of clinically used doses of fluconazole, griseofulvin, itraconazole, ketoconazole, and terbinafine. The pattern of liver enzyme levels indicated that ketoconazole, fluconazole, and itraconazole caused mixed hepatic injury (i.e., cholestatic-hepatocellular injury) while griseofulvin and terbinafine appear to have predominantly resulted in cholestatic injury. Azole antifungal agents have been reported to cause both hepatocellular and cholestatic injury [18]. The increase in liver enzymes with longer treatment duration was noted with all antifungal agents, with itraconazole and terbinafine recording the highest changes in liver enzymes. In contrast, significant histological changes were observed with itraconazole while only slight worsening in hepatic damage was observed with ketoconazole treatment. The increase in the risk of hepatotoxicity during longer treatment with antifungal agents has been reported in several studies [22–24, 31] and regular monitoring of liver enzymes in patients that require long treatment with antifungal agents is standard practice.

Based on liver enzyme levels observed in this study, ketoconazole had the highest risk in causing liver injury followed by itraconazole, fluconazole, terbinafine, and griseofulvin. The relative hepatotoxicity of antifungal agents based on liver enzymes is consistent with several studies. The higher ALT levels during treatment with ketoconazole than during treatment with fluconazole observed in this study is consistent with an in vitro study that reported that ketoconazole significantly increased the levels of ALT and lactate dehydrogenase (LDH) in cultured rat hepatocytes while fluconazole had minimal effects on both biomarkers [23]. Hepatotoxicity produced by ketoconazole and its main metabolite (N-deacetyl ketoconazole) presents as elevation of ALT or lactate dehydrogenase (Rodriguez and Acosta Jr., 1997) [32, 33]. Similarly, in concordance with the present study, an in vivo study reported that itraconazole treatment resulted in significantly higher ALT and ALP levels than fluconazole in rats treated for 14 days [24]. An in vitro study using rat hepatocyte cultures also reported similar findings regarding the relative hepatotoxicity of itraconazole and fluconazole [34]. More recently, a meta-analysis of 39 studies incorporating more than 8,000 patients reported that 17.4% of patients treated with itraconazole had elevated serum liver enzymes compared to 2.0% of fluconazole users [19]. Griseofulvin has also been observed to have a lower risk of causing hepatotoxicity than ketoconazole in clinical studies [11].

The observation that fluconazole causes more hepatic damage than ketoconazole based on histological examinations is not consistent with an in vitro study which reported that ketoconazole caused more hepatotoxicity than fluconazole in cultured rat hepatocytes (Rodriguez and Acosta Jr. 1995) [23]. Another in vitro study reported that itraconazole caused more hepatic damage than fluconazole in rat hepatocyte cultures while the present study observed that fluconazole causes more hepatic damage than itraconazole based on histology examinations [34]. Similarly, in an in vivo study by Somchit et al. (2004), hepatocellular necrosis, degeneration of periacinar and midzonal hepatocytes, bile duct hyperplasia, biliary cirrhosis, and giant cell granuloma were observed in rats treated with itraconazole while mild degenerative changes of centrilobular hepatocytes were observed in the rats treated with fluconazole [24]. However, the study by Somchit et al. (2004) used doses that ranged between 7 and 70 times higher than the recommended daily human doses in humans while the present study used doses equivalent to human therapeutic doses [24]. Similarly, the hepatocytes in the in vitro studies were exposed to doses that were higher than those used therapeutically. Therefore, the difference between observations made in the present study and the study by Somchit et al. (2004) and the in vitro studies may be explained by the differences in the hepatotoxicity mechanisms at therapeutic doses compared to toxic doses. Secondly, the different routes of administration in the present study and the study by Smochit et al. (2004) may also explain the differences in the findings. In the study by Somchit et al. (2004), drugs were administered intraperitoneally while the oral route was used in the present study [24].

The observation during histological examination that fluconazole is more hepatotoxic than ketoconazole and itraconazole is consistent with the results from a large population-based study of 90,847 users of antifungal agents in Taiwan [22]. In this Taiwanese population, the incidence rate of drug-induced liver injury in patients treated with fluconazole was more than sixfold higher than in patients treated with ketoconazole, griseofulvin, itraconazole, and terbinafine. In addition, fatality after acute liver injury was associated with fluconazole. Out of six fatal drug-induced liver injury cases, five were current users of fluconazole while one was using both fluconazole and ketoconazole [22]. In contrast to the observations made in our study using histology reports and the study by García Rodrǐguez et al. (2014), an epidemiologic study of 69,830 patients in the United Kingdom observed that the incidence rate of acute liver injury was more than 13-fold higher in patients treated ketoconazole than in patients treated with itraconazole and terbinafine [21]. In this population of patients who filled prescriptions for oral antifungal agents between 1991 and 1996, ketoconazole had the highest risk for causing acute liver injury followed by itraconazole, terbinafine, fluconazole, and griseofulvin. None of the 35,833 current users of fluconazole experienced acute liver injury while one case was associated with past use of fluconazole [21].

In the present study, biochemical assays revealed that ketoconazole was the most hepatotoxic antifungal agent while histological examinations indicated that fluconazole was the most hepatotoxic. Similarly, a population-based study that used biochemical assays as the only diagnostic tool reported that ketoconazole was the most hepatotoxic while the study that used a combination of biochemical assays, biopsy, and tissue pathology reported that fluconazole was the most hepatotoxic [21, 22]. The discrepancy between histological examinations and biochemical assays in this study and the difference between the two epidemiological studies that used different diagnostic approaches for acute liver injury suggests that relative hepatotoxicity of antifungal agents may depend on the diagnostic tests used. Furthermore, the fact that most of the drug-induced liver hepatotoxicity cases in clinical use are usually based on liver enzymes and not histological examinations may explain the higher incidence of ketoconazole-associated hepatotoxicity reports than those reported during fluconazole use. Despite their lack of specificity and poor correlation with the degree of liver injury, biochemical assays remain the cornerstone of identifying drug-induced liver injury because they are the most feasible, least-invasive, and cheapest diagnostic tests. However, given the low correlation between liver enzymes and the degree of hepatic damage [35], histology assessments provide better information in deciding the relative hepatotoxicity of chemical agents, including antifungal agents.

## 5. Conclusions

Liver enzyme levels suggested that ketoconazole is likely to cause liver injury than fluconazole while histopathological examinations revealed that fluconazole is more hepatotoxic than ketoconazole. The diagnostic criteria used in the evaluation of hepatotoxicity of antifungal agents should be taken into consideration when reviewing the evidence on their relative hepatotoxicity. Given the poor correlation between liver enzymes and the extent of liver injury, it is important to confirm liver injury through histological examination before a diagnosis of hepatotoxicity can be made in clinical settings.

## Figures and Tables

**Table 1:** Effect of the five oral antifungal agents (griseofulvin, fluconazole, ketoconazole, itraconazole, and terbinafine) on serum activities of AST, ALT, and ALP in rats after 14 days of treatment.

Groups	AST/IU mean ± SEM	ALT/IU mean ± SEM	ALP/IU mean ± SEM
Control	58.50 ± 5.40_ _^a^	60.17 ± 6.30^a^	152.17 ± 5.57^a^
Griseofulvin	124.33 ± 25.17_ _^a^	86.33 ± 8.06^a^	370.50 ± 19.81^b, c^
Fluconazole	275.83 ± 69.45_ _^a, b^	178.33 ± 42.85^a, b^	409.50 ± 29.78^b, c^
Ketoconazole	431.17 ± 101.89_ _^b^	283.17 ± 61.96^b^	324.67 ± 18.33^b^
Itraconazole	272.00 ± 48.10_ _^a, b^	172.17 ± 44.18^a, b^	414.50 ± 17.19^b, c^
Terbinafine	135.67 ± 34.98_ _^a^	88.67 ± 6.10^a^	420.33 ± 26.30^c^

AST: aspartate aminotransferase, ALT: alanine aminotransferase, and ALP: alkaline phosphatase

^a–e^Tukey's post hoc analysis: like letters (a–e) indicate nonsignificant differences.

**Table 2:** Effect of the five oral antifungal agents (griseofulvin, fluconazole, ketoconazole, itraconazole, and terbinafine) on serum activities of AST, ALT, and ALP in rats after 28 days of treatment.

Groups	AST/IU mean ± SEM	ALT/IU mean ± SEM	ALP/IU mean ± SEM
Control	58.50 ± 5.40_ _^a^	60.17 ± 6.30_ _^a^	152.17 ± 5.57_ _^a^
Griseofulvin	172.83 ± 11.48_ _^b^	114.50 ± 6.06_ _^b^	418.17 ± 8.07_ _^b^
Fluconazole	323.67 ± 19.49_ _^c^	243.00 ± 16.43_ _^c^	566.33 ± 15.46_ _^c^
Ketoconazole	608.17 ± 24.78_ _^d^	400.00 ± 14.73_ _^d^	446.00 ± 14.13_ _^b, d^
Itraconazole	440.17 ± 12.98_ _^e^	296.67 ± 13.62_ _^e^	554.33 ± 24.40_ _^c, d^
Terbinafine	179.67 ± 13.89_ _^b^	105.83 ± 4.24_ _^a, b^	499.17 ± 16.77_ _^d^

AST: aspartate aminotransferase, ALT: alanine aminotransferase, and ALP: alkaline phosphatase

^a–e^Tukey's post hoc analysis: like letters (a–e) indicate nonsignificant differences.

**Table 3:** Histopathological findings in livers of treatment with antifungal agents.

Histological findings in the liver				
Treatment group	Hepatocyte degeneration	Necrosis	Inflammation	Bile duct hyperplasia and granuloma
Ketoconazole	+++	+++	+++	++
Fluconazole	+++	+++	+++	+++
Itraconazole	++	++	++	+
Griseofulvin	+	+	+	+
Terbinafine	+	+	+	++

Normal (<4 lesions); + mild (4–7 lesions); ++ moderate (8–11 lesions); +++ severe lesions (≥12 lesions per slide); inflammation was determined based on the presence of macrophages and scattered neutrophils and eosinophils in central venules.
